# Comparison of antibacterial effects of orthodontic composites containing different nanoparticles on Streptococcus mutans at different times

**DOI:** 10.1590/2177-6709.25.2.052-060.oar

**Published:** 2020

**Authors:** Soghra Yassaei, Ali Nasr, Hengameh Zandi, Mohammad Nima Motallaei

**Affiliations:** 1 Shahid Sadoughi University of Medical Sciences, Faculty of Dentistry, Department of Orthodontics (Yazd, Iran).; 2 Shahid Sadoughi University of Medical Sciences, School of Medicine, Department of Microbiology(Yazd, Iran).

**Keywords:** Antimicrobial, Antibacterial, Orthodontic composites

## Abstract

**Introduction:**

Plaque accumulation can cause white spot lesions. Adding nanoparticles to composites can be effective in reducing the number and function of microorganisms.

**Objective:**

The aim of this study was to evaluate the antibacterial effects of orthodontic composites containing different nanoparticles on *Streptococcus mutans* at different times.

**Methods:**

Hydroxyapatite, titanium oxides, zinc oxide, copper oxide and silver oxide nanoparticles were prepared at 0.5% and 1% weight concentrations. Accordingly, ten study groups and one control group were obtained. Then, 26 composite discs were prepared from each group. Strain of *Streptococcus mutans* was cultured, and colonies of *Streptococcus mutans* were counted. Further bacterial culture was swapped onto enriched Mueller-Hinton agar. The composites were placed on the culture medium, and after incubation the diameter of growth inhibition was measured. To investigate the long-term effect of nanoparticles, the colonies were counted at days 3, 15 and 30.

**Results:**

The results showed that 1% copper oxide and 1% silver oxide significantly reduced the number of bacteria (*p*< 0.05), but there was no significant difference between the other groups and control group (*p*> 0.05). At day three, there was a significant difference between control group and 0.5% silver oxide, 1% silver oxide and 1% copper oxide groups (*p*< 0.05). However, colonies had grown in all groups at day 30 but showed no significant difference with control group (*p*> 0.05).

**Conclusion:**

Addition of 1% copper oxide and 1% silver oxide has short-term antibacterial effects, so the clinical use of these nanoparticles cannot be justified.

## RESUMO

**Introdução:**

O acúmulo de placa bacteriana pode causar lesões de mancha branca. A adição de nanopartículas nas resinas ortodônticas pode ser eficaz para reduzir o número e a função dos microrganismos.

**Objetivo:**

O objetivo do presente estudo foi avaliar os efeitos antibacterianos contra o *Streptococcus mutans,* em diferentes intervalos de tempo*,* de resinas ortodônticas contendo diferentes tipos de nanopartículas.

**Métodos:**

Foram criados dez grupos experimentais e um grupo controle contendo nanopartículas de hidroxiapatita, óxido de titânio, óxido de zinco, óxido de cobre e óxido de prata em concentrações de 0,5% e 1%. Em seguida, foram preparados 26 discos de resina para cada grupo. Colônias de *Streptococcus mutans* foram cultivadas e contadas. Posteriormente, as culturas bacterianas foram colocadas em solução de ágar Mueller-Hinton. Os discos de resina foram colocados no meio de cultura e, depois da incubação, mediu-se o diâmetro de inibição do crescimento. Para avaliar os efeitos em longo prazo das nanopartículas, as colônias foram analisadas após 3, 15 e 30 dias.

**Resultados:**

Os resultados mostraram que o óxido de cobre a 1% e o óxido de prata a 1% reduziram significativamente o número de bactérias (*p*< 0,05), mas não houve diferença estatisticamente significativa entre os outros grupos e o grupo controle (*p*> 0,05). Após 3 dias, houve uma diferença significativa entre o grupo controle e os grupos óxido de prata a 0,5%, óxido de prata a 1% e óxido de cobre a 1% (*p*< 0,05). Porém, após 30 dias, as colônias haviam crescido em todos os grupos, sem diferença com o grupo controle (*p*> 0,05).

**Conclusão:**

A adição de óxido de cobre a 1% e óxido de prata a 1% apresenta efeitos antibacterianos apenas no curto prazo; portanto, o uso clínico dessas nanopartículas não se justifica.

## INTRODUCTION

Decalcification of enamel surfaces adjacent to the orthodontic appliances is an important complication associated with orthodontic treatment.^1^ Despite many attempts made in line with patient health education, white spot lesions (WSL) accompanied by fixed orthodontic appliances are still a major clinical problem, and WSLs have been increasing since the advent of bonding brackets.^2^ These spots can apparently lead to patient dissatisfaction after orthodontic treatment.^2^

The first step in preventing WSL is achieving appropriate oral health, including tooth brushing and fluoride toothpaste. For patients with poor cooperation, use of antimicrobial bonding systems around the brackets is helpful. Use of resin-modified glass ionomers (RMGIs), fluoride varnishes and ACP (Amorphous Calcium Phosphate) is also effective in preventing caries.^3^ ^,^ ^4^ Various studies have shown that more plaque is accumulated around composites compared with other restorative materials or hard dental tissue, which leads to more secondary caries around resin composite restorations.^2^ ^,^ ^5^ ^,^ ^6^ This can occur due to surface roughness and the energy released from these materials, which can be caused by the type of resin, size of filler and percentage of filler in the composites.^2^ ^,^ ^5^ ^,^ ^6^ Moreover, none of the components of resin composites has bacteriostatic properties. That is why new studies have drawn a special attention to the antibacterial properties of resin composites to reduce the risk of recurrent caries around direct composite restorations.^1^ ^,^ ^2^ ^,^ ^4^

Various approaches have been adopted to add antibacterial properties to resin composites and adhesives. The first approach is adding antibacterial materials to the resin matrix that are released over time and inhibit bacterial growth. Examples of this class are addition of materials such as fluoride and chlorhexidine. Although they have initially powerful antibacterial properties, their release does not last for a long time. In addition, the composites having these materials and many others of the same kind have a higher rate of bond failure due to the adverse effects of these materials on their mechanical characteristics.^7^ ^,^ ^8^ The second approach is adding quaternary ammonium to resin monomers. It seems that this method would be more successful because antibacterial properties last for a longer time.^9,10^ The third approach is adding metal/metal oxides as particles or ions to restorative materials. For many years metals such as silver, gold and zinc have been used as bactericidal and bacteriostatic materials. The antibacterial properties of metals are directly influenced by their surface area. The dimensions of nanoparticles allow more interaction with microorganisms, thereby increasing their antibacterial properties.^11^

*Streptococcus mutans* is one of the main bacteria responsible for caries. Some studies have proposed silver nanoparticles as the most effective type of metals among metal nanoparticles for preventing the growth of *Streptococcus mutans*.^12^ ^,^ ^13^ In addition to silver, many other nanoparticles like hydroxyapatite, chitosan, copper acids, titanium, zinc and silicone dioxide (SiO_2_) have been added to composites and have been investigated.^1^ ^,^ ^2^ ^,^ ^14^ ^-^ ^16^ This study was conducted to compare the antibacterial effects of adding different nanoparticles to orthodontic composites on the growth of *Streptococcus mutans* at different times. 

## MATERIAL AND METHODS

### Nanocomposite preparation

**Figure 1:**
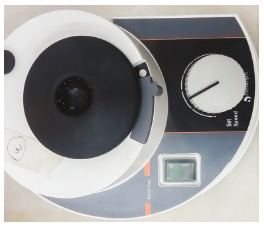
Vortex machine.

This study was approved by the local Ethical Committee of Shahid Sadoughi University, with the reference number IR.SSU.REC.1396.60, on Feb 2017. In this study, hydroxyapatite (Aldrich), titanium oxides (Sigma-Aldrich), zinc (Aldrich), copper (Aldrich) and silver (Merck) nanoparticles were prepared at 0.5% and 1% weight concentrations, measured by a digital scale with four decimal places, and were mixed with light cure orthodontic composite (3M Unitek, Monrovia, California, USA, Transbond XT) in a semi-dark environment using a mixer spatula and a glass slab.^2^ ^,^ ^16^ Hence, ten study groups and one control group (without nanoparticles) were obtained. For 0.5% groups (five groups), 0.0065 g of nanoparticles and 1.2935 g of composite were used, and for 1% groups (five groups), 0.013 g of nanoparticles and 1.287 g of composite were used. Each group was mixed in a vortex machine (Heidolph, Germany) (Fig 1) for five minutes. To prevent water penetration into the nanoparticle composites, they were placed in previously sterilized capped test tubes, and to prevent light transmission to the composites, the test tubes were insulated with a black Teflon tape and were then placed in Sonicator machine(Elma D-78224) (Fig 2) for 60 minutes. To prevent the temperature rise of composites, ice was added to the water in the machine, to keep the temperature stable. Then, 26 composite discs with 6-mm diameter and 1-mm thickness were prepared for each group (ten study groups and one control group). Gamma ray was applied to sterilize the samples. 

**Figure 2:**
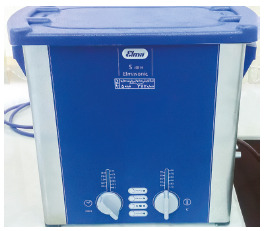
Sonicator machine.

### Antimicrobial test

To prepare fresh bacterial medium, *Streptococcus mutans* strain ATCC25175^2^ ^,^ ^14^ was prepared from the Pasteur Institute, was then inoculated onto the culture medium according to the manufacturer’s instructions, and incubated at 37°C in a CO_2_ incubator for 24 h.^14^ After incubation, the fresh medium was transferred to sterile 5-mm test tubes containing physiologic serum, and 0.5 McFarland turbidity standard was prepared.

### Biofilm inhibition

Sterile TSB culture medium was used to determine the adhesion. To this end, a composite sample, 1.5 ml TSB culture medium and 0.1 ml bacterial suspension were placed in each well. The plates were incubated at 37°C for 24 h in a CO_2_ incubator, to separate the bacteria attached to the composite (Fig. 2-1). The samples were then transferred to the test tube containing 3 ml physiologic serum and then in an ultrasonic bath at a frequency of 25 Hz to isolate the biofilm from the composite. Next, 10^-1^ to 10^-4^ dilutions were prepared from the suspension obtained in sterile physiologic serum. To count the number of bacteria in the prepared dilutions, 0.1 ml of the suspension was inoculated onto the BHI agar plate, cultured and incubated at 37°C in the CO_2_ incubator for 48 h. Then, the *Streptococcus mutans* colonies in the plates were counted by a colony counter machine, and CFU/ml of the bacteria was determined. Given the dilution coefficient, the number of bacteria in 1 mL was determined and calculated in log10^2^.

**Figure 2-1:**
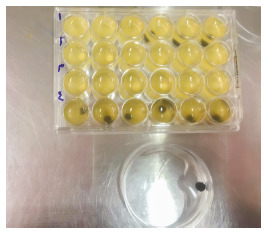
Microplates and washed composite disk after getting out of the well.

### Disc agar diffusion (DAD) test

Next, 0.5 McFarland turbidity standard was prepared from the pure and fresh bacterial culture in the test tube containing physiologic serum. It was then swapped onto the Mueller-Hinton agar enriched with 5% sheep blood and cultured afterwards. The composites containing 0.5% and 1% concentrations were cultured onto the culture medium, and after incubation at 37°C for 24 h, the diameter (mm) of growth inhibition was measured by a ruler. Penicillin and physiologic serum were used for the sake of control.^2^

### Antibacterial properties of eluted components

To assess the irrigated materials, the discs containing nanoparticle in the 5-ml BHI broth test tubes were used in a dark environment and temperature of 37°C (Fig. 2-2). At days 3, 15 and 30, the discs were removed from the culture medium, and the materials were transferred to sterile test tubes. Then, 50 µl bacterial suspension with 2.5 × 10^5^ concentration were added to the new tubes and incubated in a shaking incubator for 24 h. It was then transferred from the test tubes to the blood agar, and the number of bacteria was counted.^2^ All culture media used in this study were made by Liofilchem, Italy.

**Figure 2-2:**
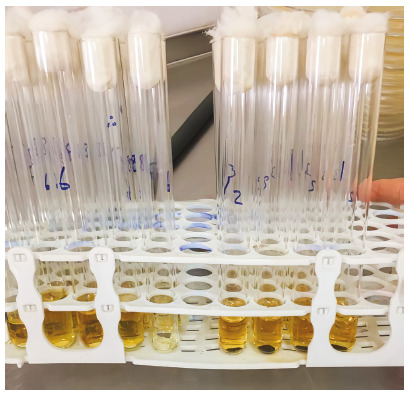
Disks containing nanoparticles in tubes with 5 ml of BHI broth.

### Statistical analysis

Data were fed into SPSS-22 software, and normality of data was analyzed by Kolmogorov-Smirnov test. Given the normality of data, ANOVA test was used for comparison of the groups and Tukey-HSD test was used for pair comparison of groups. Further, chi-square test was used to compare the study groups at different times. 

## RESULTS

This study compared the antibacterial effect of 0.5% and 1% concentrations of copper oxide, silver oxide, zinc oxide, hydroxyapatite and titanium oxide nanoparticles. ANOVA test was run to compare the study groups (Table 1). Then, Tukey-HSD test was used for pair comparison of groups. The findings showed a significant difference between 1% copper oxide group and all other groups, except for 1% silver oxide group. Further, there was a significant difference between 1% silver oxide group and all other groups, except 1% copper oxide group (*p*< 0.05). There were no significant differences between other groups and control group (*p*> 0.05) (Table 2). 

**Table 1:** Mean (SD) of *Streptococcus mutans* colonies in the study groups.

Group	n	Mean	SD
CuO 0,5%	6	266.67	40.825
ZnO 0,5%	6	283.33	25.82
HA 1%	6	300	0
AgO 0,5%	6	275	27.386
HA 0,5%	6	300	0
Control	6	300	0
CuO 1%	6	158.33	58.452
TiO_2_ 1%	6	241.67	66.458
AgO 1%	6	133.33	68.313
TiO_2_ 0,5%	6	300	0
ZnO 1%	6	291.67	20.412
Total	66	259.09	66.742

**Table 2:** Comparison of mean difference between study groups in biofilm-inhibition test, using Tukey-HSD test.

Groups	ZnO 1%	TiO_2_ 0.5%	AgO 1%	TiO_2_ 1%	CuO 1%	Control	HA 0.5%	AgO 0.5%	HA 1%	ZnO 0.5%	CuO 0.5%
CuO 0.5%	-25	-33.3	133.3	25	108.3	33.3	33.3	-8.3	33.3	16.6	-
*p-valor*	0.987	0.909	<0.001	0.987	<0.001	0.909	0.909	1	0.909	1	
ZnO 0.5%	-8.3	-16.6	150	41.6	125	-16.6	-16.6	8.3	-16.6	-	16.6
*p-valor*	1	1	<0.001	0.719	<0.001	1	1	1	1		1
HA 1%	8.3	0	166.6	85.3	141.6	0	0	25	-	-16.6	33.3
*p-valor*	1	1	<0.001	0.25	<0.001	1	1	0.987		1	0.909
AgO 0.5%	-16.6	-25	141.6	33.3	116.6	-25	-25	-	25	8.3	-8.3
*p-valor*	1	0.987	<0.001	0.909	<0.001	0.987	0.987		0.987	1	1
HA 0.5%	8.3	0	166.6	58.3	141.6	0	-	-25	0	-16.6	33.3
*p-valor*	1	1	<0.001	0.25	<0.001	1		0.987	1	1	0.909
Control	8.3	0	166.6	58.3	141.6	-	0	-25	0	-16.6	33.3
*p-valor*	1	1	<0.001	0.25	<0.001		1	0.987	1	1	0.909
CuO 1%	133.3	-141.6	25	-83.3	-	141.6	141.6	116.6	141.6	125	108.3
*p-valor*	<0.001	<0.001	0.987	0.015		<0.001	<0.001	<0.001	<0.001	<0.001	<0.001
TiO_2_ 1%	-50	-58.3	108.3	-	-83.3	58.3	58.3	33.3	58.3	41.6	25
*p-valor*	0.468	0.25	<0.001		0.015	0.25	0.25	0.909	0.25	0.719	0.987
AgO 1%	158.3	-166.6	-	108.3	25	166.6	166.6	141.6	166.6	150	133.3
*p-valor*	<0.001	<0.001		<0.001	0.987	<0.001	<0.001	<0.001	<0.001	<0.001	<0.001
TiO_2_ 0.5%	8.3	-	-166.6	-58.3	-141.6	0	0	-25	0	-16.6	-33.3
*p-valor*	1		<0.001	0.25	<0.001	1	1	0.987	1	1	0.909
ZnO 1%	-	8.3	158.3	-50	133.3	8.3	8.3	-16.6	8.3	-8.3	-25
*p-valor*		1	<0.001	0.468	<0.001	1	1	1	1	1	0.987

Chi-square test was used to compare each group at different times. As shown in Table 3, 0.5% and 1% silver oxide showed lack of colony growth at day 15, but the difference was not statistically significant (*p*> 0.05). In this study, disc diffusion test was used, too. For this purpose, eight samples were included in each group. The mean, standard deviation and diameter of growth inhibition are shown in Table 4. Tukey-HSD test was applied for pair comparison of study groups. As indicated in Table 5, there were significant differences between 1% copper oxide and 1% silver oxide groups and other study groups (*p*< 0.05), but no significant difference was found between 1% silver oxide and 1% copper oxide groups (*p*> 0.05), which is in line with the results obtained for the inhibitory effect of nanoparticles on the number of colonies (Table 5). 

**Table 3:** Comparison of study groups at days 3, 15 and 30.

	Day					
	3		15		30	
	*Success* *percentage*	*Repeat* *number*	*Success* *percentage*	*Repeat* *number*	*Success* *percentage*	*Repeat* *number*
CuO 0.50%	0	3	0	3	0	3
ZnO 0.50%	0	3	0	3	0	3
HA 1%	0	3	0	3	0	3
AgO 0.50%	0	3	33.3	3	66.7	3
HA 0.5%	0	3	0	3	0	3
Control	0	3	0	3	66.7	3
CuO 1%	0	3	0	3	0	3
TiO_2_ 1%	0	3	0	3	0	3
AgO 1%	0	3	66.7	3	100	3
TiO_2_ 0.5%	0	3	0	3	0	3
ZnO 1%	0	3	0	3	0	3

**Table 4:** Mean (SD) of the zone of inhibition diameter in the study groups.

Groups	n	Mean	SD
CuO 0.5%	8	7.5	0.756
ZnO 0.5%	8	6	0
HA 1%	8	6	0
AgO 0.5%	8	8.5	0.535
HA 0.5%	8	6	0
Control	8	6	0
CuO 1%	8	8	0.535
TiO_2_ 1%	8	6	0
AgO 1%	8	9.13	0.641
TiO_2_ 0.5%	8	6	0
ZnO 1%	8	6	0
Total	88	6.83	1.215

**Table 5:** Comparison of mean difference between study groups in the zone of inhibition diameter, using Tukey-HSD test.

Groups	ZnO 1%	TiO_2_ 0.5%	AgO 1%	TiO_2_ 1%	CuO 1%	Control	HA 0.50%	AgO 0.5%	HA 1%	ZnO 0.50%	CuO 0.50%
CuO 0.5%	1.5	1.5	-1.625	1.5	-0.5	1.5	1.5	-1	1.5	1.5	--
*p-valor*	<0.001	<0.001	<0.001	<0.001	0.237	<0.001	<0.001	<0.001	<0.001	<0.001	
ZnO 0.5%	0	0	-3.125	0	-2	0	0	2.5	0	--	1.5
*p-valor*	1	1	<0.001	1	<0.001	1	1	<0.001	1		<0.001
HA 1%	0	0	-3.125	0	-2	0	0	-2.5	--	0	1.5
*p-valor*	1	1	<0.001	1	<0.001	1	1	<0.001		1	<0.001
AgO 0.5%	2.5	2.5	-0.625	2.5	0.5	2.5	2.5	--	-2.5	2.5	-1
*p-valor*	<0.001	<0.001	0.049	<0.001	0.237	<0.001	<0.001		<0.001	<0.001	<0.001
HA 0.5%	0	0	1.2503	0	-2	0	--	2.5	0	0	1.5
*p-valor*	<0.001	1	<0.001	1	<0.001	1		<0.001	1	1	<0.001
Control	0	0	-3.125	0	-2	--	0	2.5	0	0	1.5
*p-valor*	1	1	<0.001	1	<0.001		1	<0.001	1	1	<0.001
CuO 1%	2	2	-1.125	2	--	-2	-2	0.5	-2	-2	-0.5
*p-valor*	<0.001	<0.001	<0.001	<0.001		<0.001	<0.001	0.237	<0.001	<0.001	0.237
TiO_2_ 1%	0	0	-3.125	--	2	0	0	2.5	0	0	1.5
*p-valor*	1	1	<0.001		<0.001	1	1	<0.001	1	1	<0.001
AgO 1%	3.125	3.125	--	-3.125	-1.125	-3.125	1.2503	-0.625	-3.125	-3.125	-1.625
*p-valor*	<0.001	<0.001		<0.001	<0.001	<0.001	<0.001	0.049	<0.001	<0.001	<0.001
TiO_2_ 0.5%	0	--	3.125	0	2	0	0	2.5	0	0	1.5
*p-valor*	1		<0.001	1	<0.001	1	1	<0.001	1	1	<0.001
ZnO 1%	--	0	3.125	0	2	0	0	2.5	0	0	1.5
*p-valor*		1	<0.001	1	<0.001	1	1	<0.001	1	1	<0.001

## DISCUSSION

In the present study, copper oxide, silver oxide, zinc oxide, hydroxyapatite and titanium oxide nanoparticles were added to composites at 0.5% and 1% concentrations, and antimicrobial effects of the composites containing these nanoparticles on *Streptococcus mutans* were compared at different times. Comparison of the effects of these nanoparticles at different concentrations at different times in one study is one of the advantages of the present study. Some studies have used higher concentrations of nanoparticles.^17^ ^,^ ^18^ The highest concentration of nanoparticles used in the current study was 1%. Higher concentrations may cause toxicity and esthetic problems in cases like silver oxide. In addition, studies have shown that use of nanoparticles such as 1% zinc oxide and silver oxide can significantly increase antibacterial effects without reducing mechanical properties like shear strength, shear coefficient, compressive strength and shear bond strength of resin composites.^18^ ^-^ ^20^ Use of low concentrations of metal nanoparticles can decrease severe discoloration and esthetic problems of resin composites.^14^ The results of the present study indicated a significant difference between 1% copper oxide and silver oxide and other study groups, including control group. However, no significant difference was found between 1% copper oxide and silver oxide nanoparticles. It should be noted that the curing time increased in copper oxide and silver oxide groups, which could be due to light absorption by these nanoparticles due to their black color. Both copper and silver oxides have been coated on and incorporated with other materials.^21^ Copper oxide is cheaper and chemically and physically more stable than silver. Copper oxide nanoparticles in suspensions have shown extensive antimicrobial effects against a wide range of pathogen bacteria. One of the most important mechanisms of antimicrobial effects of nanoparticles is destruction of the membrane of microorganisms. Since *Streptococcus mutans* is a gram-positive bacterium, copper nanoparticles seem to affect this bacterium.^12^ ^,^ ^21^

Some studies suggested the incorporation of copper oxide nanoparticles with polymers, the same as silver.^22^ ^,^ ^23^ Argueta-Figueroa et al.^24^ conducted a study to investigate the antimicrobial effects of composites containing copper nanoparticles. They added nanoparticles at weight concentrations of 0.0100%, 0.0075% and 0.0050% and concluded that the composites containing copper nanoparticles had a significant inhibitory effect on the studied microbes. Poosti et al.^4^ carried out a study on the antibacterial effects of copper oxide and zinc oxide nanoparticles coated on orthodontic brackets and reported that copper and copper-zinc nanoparticles had antimicrobial effect on *Streptococcus mutans*. Moreover, Toodehzaeim et al.^16^ studied the antimicrobial effects of adhesives containing copper oxide nanoparticles and reported antimicrobial effects for these materials. Hence, the results of the present study are in agreement with those of Argueta-Figueroa et al.^24^ and Toodehzaeim et al.^16^

Silver oxide was a nanoparticle that showed a significant effect in inhibition of *Streptococcus mutans* growth in the current study. Silver oxide has a good antibacterial activity and its surface/volume ratio and antibacterial activity are increased when it is converted to nanoparticle. This substance has a potent cytotoxic effect on a wide range of microorganisms in both ionic and metallic forms. Several studies have reported the cytotoxicity of silver nanoparticles on the fungi, protozoa, a number of viruses and gram-positive and -negative bacteria like *Streptococcus mutans*, lactobacillus, *Escherichia coli* and *Staphylococcus aureus,* as well as antibacterial and bactericidal properties. Since silver affects *Streptococcus* in oral cavity and periodontal pathogens and inhibits bacterial adhesion to the surfaces as well as biofilm formation, it can be added to the dental materials as a useful antibacterial additive.^3^ ^,^ ^12^ ^,^ ^13^ ^,^ ^15^ Some studies have reported the antibacterial activity of silver nanoparticles in tooth-colored restoration against oral *Streptococcus*.^12^ ^,^ ^13^

It is believed that silver ions interfere with three major components of the bacterial cell wall, including peptidoglycan cell wall, plasma membrane and bacterial DNA, as well as bacterial proteins, especially the enzymes involved in vital cell processes such as electron transfer chain, and cause cell death. It has also been reported that the silver ions lyse bacterial cell wall.^12^ Yet, there are concerns about silver toxicity which have limited the use of silver in human being, so they need to be taken into account.^25^ Moreover, nanotechnology has made possible the production of silver nanoparticles with a smaller size and higher surface/volume ratio, which can produce higher antimicrobial effect and less toxicity in human.^15^

Kasraei et al.^26^ carried out a study on the antimicrobial effects of resin composites containing silver nanoparticles on *Streptococcus mutans* and *lactobacillus*. The findings showed the silver nanoparticle composites had a higher antimicrobial effect, compared to control group, confirming the results of the present study. The review study by Bapat et al.^12^ proposed silver nanoparticles as very good nanoparticles to be added to different materials due to having excellent antimicrobial properties and not affecting the mechanical properties of the materials.^12^ Another study by Chambers et al.^27^ showed that adding Ag-TiO_2_ to orthodontic polymers induced antimicrobial effects.

Another nanoparticle studied in the present research was titanium oxide. The results showed 1% titanium oxide reduced the number of colonies but presented no significant difference with control group, which was in contrast with the findings of the study by Poosti et al.^4^ It should be noted that spontaneous curing of composite was observed in titanium oxide group. Mirhashemi et al.^28^ conducted a study on the antimicrobial effects of adding zinc oxide and chitosan nanoparticles on orthodontic composites. The antimicrobial effects of these nanoparticles on the growth of *Streptococcus mutans*, *Streptococcus sanguinis* and *Lactobacillus acidophilus* as both planktonic and biofilm on the composites were investigated in four groups. Three groups with 1%, 5% and 10% concentrations of nanoparticles and one control group were also considered. The findings showed that nanoparticles could add significant antimicrobial effects to the composite only at 10% concentration. In the study of Mirhashemi et al.^28^, higher concentrations such as 5 and 10% were used. In the given study, zinc oxide showed an inhibitory diameter of zero even at 10% concentration. Poosti et al.^4^ performed a study on the antibacterial effects of copper oxide and zinc oxide nanoparticles coated on orthodontic brackets, and concluded that the antibacterial effects of copper and copper-zinc nanoparticles on *Streptococcus mutans* were higher than those of zinc nanoparticle. In the present study as well, there was no significant difference between the control and zinc oxide groups.

Abdulkareem et al.^29^ investigated the anti-biofilm effect of hydroxyapatite and zinc oxide nanoparticles and found that use of hydroxyapatite was not effective. In the present study, there was no significant difference between hydroxyapatite and control groups, and hydroxyapatite indicated a lower effect than zinc. Hence, the results of the present study are in agreement with those of Abdulkareem et al. ^29^, Mirhashemi et al.^28^ and Poosti et al.^4^

Considering the long-term presence of these composites in the patients, it is necessary to consider the long-term effect of their antibacterial properties to justify their clinical application. A significant point in this study was the analysis of antibacterial effect of these nanoparticles at days 3, 15 and 30, in order to compare their long-term effects, something which has not been investigated in similar studies.^14^ ^,^ ^24^ ^,^ ^26^ However, the results of the present study indicated that 1% copper oxide and 1% silver oxide and 0.5% silver oxide groups showed antibacterial effects at day 3. Also, 0.5 and 1% silver oxide nanoparticles showed lack of colony growth at day 15, but there was no statistically significant difference between them. However, none of the study groups showed antibacterial effects at day 30. This questions the clinical use of these nanoparticles. Furthermore, Poosti et al.^4^ studied the 30-day effect of 1% titanium oxide and reported its long-term effects during this period. But the present research showed none of these effects in all periods. Thus, further studies are needed to determine the periods in which composite nanoparticles exert their effects. In addition, some important issues like long-term clinical application and bond strength of brackets to these composites should be explored. 

## CONCLUSION

Adding 1% copper oxide and 1% silver oxide provides short-term antibacterial effects, which, however, does not justify their clinical use.
